# 2,2′-Oxybis[1,3-bis­(4-meth­oxy­phen­yl)-2,3-di­hydro-1*H*-benzo[*d*][1,3,2]di­aza­borole]

**DOI:** 10.1107/S2414314620012481

**Published:** 2020-09-18

**Authors:** Hannah H. Mallard, Nicholas D. Kennedy, Nathan A. Rudman, Alexa M. Greenwood, Jonathan Nicoleau, Corey E. Angle, Nicole A. Torquato, Michael R. Gau, Patrick J. Carroll, Mitchell R. Anstey

**Affiliations:** a Department of Chemistry, Davidson College, Davidson, North Carolina, USA; b Department of Chemistry, University of Pennsylvania, Philadelphia, Pennsylvania, 19104-6323, USA; c Department of Chemistry and Biochemistry, University of California San Diego, La, Jolla, California, USA; University of Aberdeen, Scotland

**Keywords:** crystal structure, bridging μ-oxo, boron

## Abstract

In the title compound, C_40_H_36_B_2_N_4_O_5_, the B—O—B bond angle is 132.75 (13) and the dihedral angle between the benzodiazborole rings is 73.02 (5)°. In the crystal, weak C—H⋯O inter­actions link the mol­ecules.

## Abstract 2

The title compound features a B—O—B bond angle of 132.75 (13)°.

## Graphical abstract

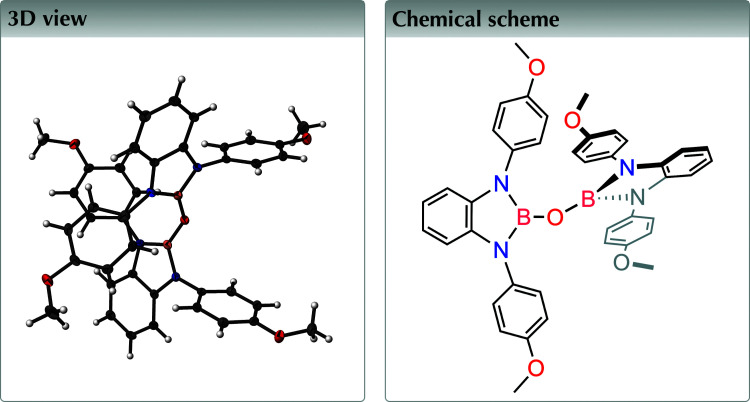

## Structure description

The field of cooperative catalysis has given scientists the ability to access more complex mol­ecular transformations using cheaper, readily available metals (Allen *et al.*, 2012 ▸; Lohr & Marks, 2015 ▸). The title compound, C_40_H_36_B_2_N_4_O_5_, was synthesized using elements from the main group of the periodic table, which are cheaper and more accessible than the traditionally used transition metals (Karunananda *et al.*, 2017 ▸; Power, 2010 ▸).

**Figure 1:**
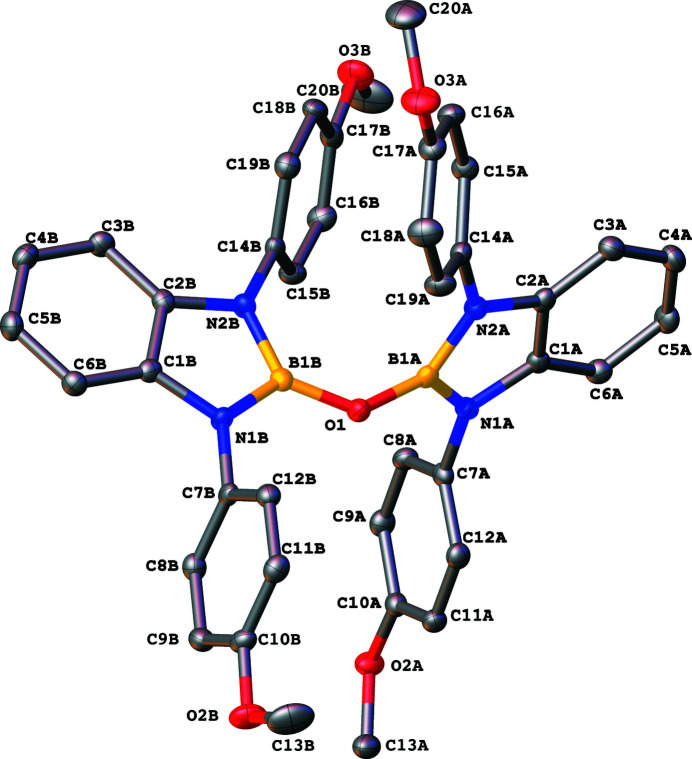
The mol­ecular structure of the title compound. Hydrogen atoms have been omitted for clarity. Ellipsoids are at 50% probability.

The title compound has a pincer-like orientation formed by an oxygen single-atom bridge connected to two Lewis-acidic boron centers (Fig. 1 ▸). The di­amine moieties bound to the boron atoms provide redox-active sites, which give the structure the electron equivalents that boron lacks while also modulating the steric environment (Prier *et al.*, 2013 ▸; Pye *et al.*, 2017 ▸; Bellemin-Laponnaz *et al.*, 2014 ▸). The pincer shape might allow the compound to use the boron atoms and the redox-active ligands to create a binding pocket for coordination and bridging of a small mol­ecule substrate.

The B1*A*—O1—B1*B* bond angle is 132.75 (13)°, which is reasonable given the steric bulk that is present in the di­aza­borole moiety. Additionally, it is likely that a *p*-type electronic inter­action exists between O1 and the adjacent boron atoms (B1*A* and B1*B*) that would serve to open up the bond angle substanti­ally beyond the textbook angle of 109.5° for an O atom bearing two lone pairs of electrons. As a result of steric encumbrance, the B1*A* and B1*B* benzodi­aza­borole rings are angled away from one another to a near perpendicular orientation, with a plane-to-plane tilt of 73.02 (5)°. The dihedral angles between the B1*A* benzodi­aza­borole ring system and its pendant *p*-meth­oxy­benzene rings are 80.49 (6) and 49.84 (7)° for the C7*A* and C14*A* rings, respectively. Comparable data for the B1*B* ring system and its pendant C7*B* and C14*B* rings are 78.32 (6) and 65.96 (7)°, respectively. The C atoms of the meth­oxy groups are all close to their respective ring planes: C13*A* [deviation = 0.333 (2) Å]; C20*A* [0.254 (2) Å]; C13*B* [−0.040 (2 Å)]; C20*B* [0.193 (2) Å].

In the crystal, weak C—H⋯O inter­actions (Table 1 ▸) link the mol­ecules.

## Synthesis and crystallization

**Figure 2:**
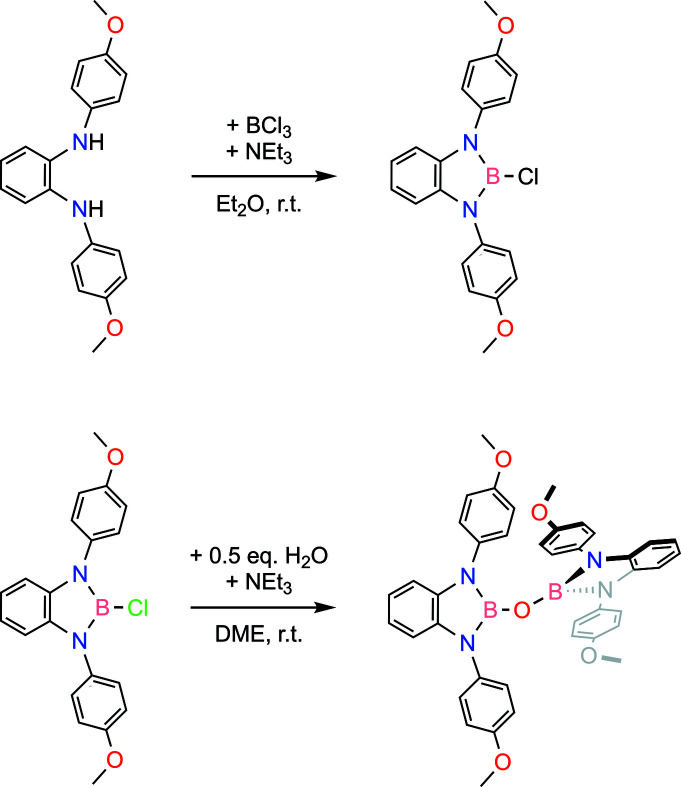
Chemical scheme for the synthesis of the title compound.

The title compound was synthesized in two steps (Fig. 2 ▸) from the previously reported precursor, *N* ^1^,*N* ^2^-bis­(4-meth­oxy­phen­yl)benzene-1,2-di­amine (Xiong *et al.*, 2018 ▸; Wang *et al.*, 2018 ▸).

Under an anhydrous nitro­gen atmosphere, 12 mmol of the di­amine precursor was dissolved in 400 ml of diethyl ether. An excess of tri­ethyl­amine, four equivalents, was then added. A stoichiometric amount of boron trichloride was added to this stirred solution whereupon a white precipitate composed of a mixture of tri­ethyl­ammonium chloride and the monomeric di­aza­borole chloride was formed. The volatiles were removed under reduced pressure to give a white solid. The solid was extracted in a fritted glass filter with a minimum volume of benzene, and the filtrate was evaporated under reduced pressure to give the crude di­aza­borole chloride. This crude solid was recrystallized from a toluene/hexa­nes mixture. The di­aza­borole chloride, (II), was obtained in 87% yield. The single-crystal X-ray structure of the di­aza­borole chloride has been deposited with the Cambridge Structural Database (Mallard *et al.*, 2020 ▸).

Under an anhydrous nitro­gen atmosphere, a solution was prepared that contained 3.0 mmol of (II), four equivalents of tri­ethyl­amine, and ∼200 ml of 1,2-di­meth­oxy­ethane. This solution was then treated with half an equivalent of water (used as a 1 *M* solution in 1,2-di­meth­oxy­ethane). After stirring overnight, a white precipitate of the tri­ethyl­ammonium chloride formed that was then filtered and discarded. The filtrate was dried under reduced pressure to give the crude product. The solid was extracted in a fritted glass filter with a minimum volume of benzene, and the filtrate was evaporated under reduced pressure to give the title compound in 85% yield.

Single crystals suitable for X-ray analysis were obtained from a saturated solution of hexa­nes. The solution was allowed to stand overnight whereupon small colorless crystals formed.

## Refinement

Crystal data, data collection and structure refinement details are summarized in Table 2 ▸. A small number of intense low-angle reflections are missing from this data set due to the arrangement of the instrument with a conservatively sized beam stop. The large number of reflections in the data set ensures that no particular bias has been introduced.

## Supplementary Material

Crystal structure: contains datablock(s) global, I. DOI: 10.1107/S2414314620012481/hb4361sup1.cif

Structure factors: contains datablock(s) I. DOI: 10.1107/S2414314620012481/hb4361Isup2.hkl

Supporting information file. DOI: 10.1107/S2414314620012481/hb4361Isup4.cml

CCDC reference: 2031384

Additional supporting information:  crystallographic information; 3D view; checkCIF report

## Figures and Tables

**Table 1:** Hydrogen-bond geometry (Å, °)

*D*—H⋯*A*	*D*—H	H⋯*A*	*D*⋯*A*	*D*—H⋯*A*
C8*B*—H8*B*⋯O2*A* ^i^	0.95	2.40	3.233 (2)	147
C13*B*—H13*E*⋯O3*B* ^ii^	0.98	2.46	3.374 (3)	155

Symmetry codes: (i) 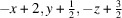 ; (ii) 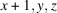 .

**Table 2:** Experimental details

Crystal data	
Chemical formula	C_40_H_36_B_2_N_4_O_5_
*M*_r_	674.35
Crystal system, space group	Monoclinic, *P*2_1_/*c*
Temperature (K)	100
*a*, *b*, *c* (Å)	16.7584 (15), 13.6696 (14), 16.0291 (17)
β (°)	111.125 (5)
*V* (Å^3^)	3425.2 (6)
*Z*	4
Radiation type	Mo *K*α
μ (mm^−1^)	0.09
Crystal size (mm)	0.17 × 0.07 × 0.05

Data collection	
Diffractometer	Bruker D8QUEST
Absorption correction	Multi-scan (*SADABS*; Bruker, 2016 ▸)
*T*_min_, *T*_max_	0.696, 0.745
No. of measured, independent and observed [*I* > 2σ(*I*)] reflections	46461, 6305, 4773
*R*_int_	0.063
(sin θ/λ)_max_ (Å^−1^)	0.604

Refinement	
*R*[*F* ^2^ > 2σ(*F* ^2^)], *wR*(*F* ^2^), *S*	0.039, 0.093, 1.02
No. of reflections	6305
No. of parameters	464
H-atom treatment	H-atom parameters constrained
Δρ_max_, Δρ_min_ (e Å^−3^)	0.19, −0.23

Computer programs: *APEX2* and *SAINT* (Bruker, 2016 ▸), *olex2.solve* (Bourhis *et al.*, 2015 ▸), *SHELXL2018/3* (Sheldrick, 2015 ▸) and *OLEX2* (Dolomanov *et al.*, 2009 ▸).

## full crystallographic data

### Crystal data

**Table:**

C_40_H_36_B_2_N_4_O_5_	*F*(000) = 1416
*M**_r_* = 674.35	*D*_x_ = 1.308 Mg m^−^^3^
Monoclinic, *P*2_1_/*c*	Mo *K*α radiation, λ = 0.71073 Å
*a* = 16.7584 (15) Å	Cell parameters from 9875 reflections
*b* = 13.6696 (14) Å	θ = 2.6–25.3°
*c* = 16.0291 (17) Å	µ = 0.09 mm^−^^1^
β = 111.125 (5)°	*T* = 100 K
*V* = 3425.2 (6) Å^3^	Plank, clear colourless
*Z* = 4	0.17 × 0.07 × 0.05 mm

### Data collection

**Table:**

Bruker D8QUEST diffractometer	4773 reflections with *I* > 2σ(*I*)
ω and φ scans	*R*_int_ = 0.063
Absorption correction: multi-scan (SADABS; Bruker, 2016)	θ_max_ = 25.4°, θ_min_ = 2.0°
*T*_min_ = 0.696, *T*_max_ = 0.745	*h* = −20→19
46461 measured reflections	*k* = −16→16
6305 independent reflections	*l* = −19→19

### Refinement

**Table:**

Refinement on *F*^2^	Primary atom site location: iterative
Least-squares matrix: full	Hydrogen site location: inferred from neighbouring sites
*R*[*F*^2^ > 2σ(*F*^2^)] = 0.039	H-atom parameters constrained
*wR*(*F*^2^) = 0.093	*w* = 1/[σ^2^(*F*_o_^2^) + (0.0417*P*)^2^ + 0.8558*P*] where *P* = (*F*_o_^2^ + 2*F*_c_^2^)/3
*S* = 1.02	(Δ/σ)_max_ = 0.001
6305 reflections	Δρ_max_ = 0.19 e Å^−^^3^
464 parameters	Δρ_min_ = −0.23 e Å^−^^3^
0 restraints	

### Special details

**Table:**

Geometry. All esds (except the esd in the dihedral angle between two l.s. planes) are estimated using the full covariance matrix. The cell esds are taken into account individually in the estimation of esds in distances, angles and torsion angles; correlations between esds in cell parameters are only used when they are defined by crystal symmetry. An approximate (isotropic) treatment of cell esds is used for estimating esds involving l.s. planes.
Refinement. The hydrogen atoms were treated in calculated positions and refined in the riding model approximation with distances of C—H = 0.95 and 0.98 Å for the aryl and methyl groups, respectively. Methyl group H atoms were allowed to rotate, but not to tip, in order to find the best rotameric conformation.

### Fractional atomic coordinates and isotropic or equivalent isotropic displacement parameters (Å^2^)

**Table:**

	*x*	*y*	*z*	*U*_iso_*/*U*_eq_	
O1	0.85920 (6)	0.72089 (8)	0.47878 (7)	0.0182 (3)	
O2A	0.94929 (7)	0.28970 (8)	0.67336 (7)	0.0215 (3)	
O2B	1.26822 (7)	0.80020 (9)	0.56662 (8)	0.0271 (3)	
O3B	0.46275 (7)	0.72061 (9)	0.49287 (9)	0.0293 (3)	
N2A	0.72444 (7)	0.71200 (9)	0.34165 (9)	0.0164 (3)	
N1B	0.94279 (7)	0.84989 (9)	0.58254 (9)	0.0153 (3)	
N2B	0.80077 (7)	0.83556 (9)	0.56577 (9)	0.0165 (3)	
N1A	0.77512 (8)	0.57002 (9)	0.42085 (9)	0.0165 (3)	
O3A	0.67160 (7)	1.09930 (8)	0.22224 (9)	0.0306 (3)	
C8A	0.78840 (9)	0.46361 (11)	0.54824 (11)	0.0179 (3)	
H8A	0.736024	0.488985	0.549372	0.022*	
C1B	0.92486 (9)	0.91823 (11)	0.63878 (10)	0.0155 (3)	
C4A	0.56664 (10)	0.53896 (12)	0.18616 (11)	0.0220 (4)	
H4A	0.519745	0.535105	0.130943	0.026*	
C14B	0.71442 (9)	0.80361 (11)	0.54539 (10)	0.0156 (3)	
C19B	0.64607 (10)	0.86514 (12)	0.50179 (11)	0.0196 (4)	
H19B	0.656451	0.928549	0.483657	0.024*	
C11A	0.94122 (9)	0.38913 (12)	0.54478 (11)	0.0181 (4)	
H11A	0.993502	0.363627	0.543511	0.022*	
C7B	1.02652 (9)	0.83921 (11)	0.57765 (10)	0.0153 (3)	
C12B	1.04462 (9)	0.88045 (11)	0.50785 (11)	0.0173 (3)	
H12B	1.001788	0.917154	0.463646	0.021*	
C9B	1.16985 (10)	0.77675 (12)	0.63780 (11)	0.0196 (4)	
H9B	1.213171	0.741701	0.683007	0.024*	
C2A	0.67165 (9)	0.63331 (11)	0.29705 (10)	0.0163 (3)	
C8B	1.09010 (9)	0.78759 (11)	0.64347 (11)	0.0181 (3)	
H8B	1.078610	0.759811	0.692302	0.022*	
C7A	0.82051 (9)	0.49728 (11)	0.48451 (10)	0.0160 (3)	
C12A	0.89620 (9)	0.45872 (11)	0.48261 (11)	0.0178 (3)	
H12A	0.917565	0.480128	0.438260	0.021*	
C11B	1.12469 (10)	0.86905 (12)	0.50128 (11)	0.0196 (4)	
H11B	1.136209	0.896959	0.452520	0.024*	
C10A	0.90946 (9)	0.35708 (11)	0.60870 (10)	0.0169 (3)	
C9A	0.83218 (10)	0.39372 (11)	0.60967 (11)	0.0191 (4)	
H9A	0.809716	0.370545	0.652606	0.023*	
C10B	1.18735 (9)	0.81671 (12)	0.56643 (11)	0.0190 (4)	
C2B	0.83793 (9)	0.91005 (11)	0.62841 (10)	0.0155 (3)	
C5A	0.59719 (10)	0.45422 (12)	0.23465 (11)	0.0218 (4)	
H5A	0.570709	0.393380	0.212281	0.026*	
C15B	0.69809 (9)	0.71079 (12)	0.56962 (11)	0.0191 (4)	
H15B	0.744615	0.668406	0.599301	0.023*	
C4B	0.85715 (10)	1.03368 (12)	0.73819 (11)	0.0216 (4)	
H4B	0.834773	1.074256	0.772644	0.026*	
C6A	0.66609 (9)	0.45685 (12)	0.31563 (11)	0.0184 (4)	
H6A	0.687046	0.398918	0.349096	0.022*	
C18B	0.56315 (10)	0.83459 (12)	0.48467 (11)	0.0202 (4)	
H18B	0.516732	0.877299	0.455501	0.024*	
C15A	0.62849 (10)	0.85263 (12)	0.28888 (11)	0.0195 (4)	
H15A	0.582685	0.814596	0.293193	0.023*	
C13A	1.03594 (10)	0.26680 (13)	0.68586 (11)	0.0235 (4)	
H13A	1.037828	0.231596	0.633340	0.035*	
H13B	1.060410	0.225671	0.739098	0.035*	
H13C	1.069072	0.327420	0.693708	0.035*	
C16B	0.61476 (10)	0.67826 (12)	0.55134 (11)	0.0217 (4)	
H16B	0.604205	0.613775	0.566929	0.026*	
C17B	0.54747 (9)	0.74158 (12)	0.51001 (11)	0.0197 (4)	
C17A	0.68022 (10)	1.00504 (12)	0.25366 (11)	0.0218 (4)	
C5B	0.94306 (10)	1.04153 (12)	0.74906 (11)	0.0206 (4)	
H5B	0.978511	1.086867	0.791098	0.025*	
C19A	0.77567 (10)	0.86889 (12)	0.30786 (11)	0.0223 (4)	
H19A	0.831507	0.841940	0.324531	0.027*	
C3A	0.60342 (9)	0.62980 (12)	0.21689 (11)	0.0195 (4)	
H3A	0.582056	0.687674	0.183498	0.023*	
C14A	0.70906 (9)	0.81108 (11)	0.31211 (10)	0.0169 (3)	
C3B	0.80324 (10)	0.96752 (12)	0.67782 (11)	0.0189 (4)	
H3B	0.744661	0.962050	0.670814	0.023*	
C6B	0.97784 (9)	0.98366 (11)	0.69901 (11)	0.0172 (3)	
H6B	1.036467	0.989175	0.706222	0.021*	
C1A	0.70288 (9)	0.54691 (11)	0.34553 (10)	0.0165 (3)	
B1B	0.86581 (11)	0.79655 (13)	0.53674 (12)	0.0156 (4)	
C16A	0.61347 (10)	0.94878 (12)	0.25942 (11)	0.0213 (4)	
H16A	0.557743	0.975921	0.243290	0.026*	
C18A	0.76183 (10)	0.96511 (12)	0.27972 (12)	0.0263 (4)	
H18A	0.808247	1.004032	0.278184	0.032*	
B1A	0.79043 (11)	0.67265 (13)	0.41912 (12)	0.0164 (4)	
C13B	1.28701 (12)	0.84058 (15)	0.49346 (15)	0.0377 (5)	
H13D	1.248532	0.812024	0.437283	0.057*	
H13E	1.346415	0.825729	0.500977	0.057*	
H13F	1.278949	0.911650	0.491983	0.057*	
C20A	0.59608 (11)	1.15048 (14)	0.21765 (14)	0.0363 (5)	
H20A	0.545972	1.117823	0.174811	0.055*	
H20B	0.598966	1.217911	0.198158	0.055*	
H20C	0.591353	1.150928	0.276835	0.055*	
C20B	0.44238 (12)	0.62958 (15)	0.52332 (17)	0.0465 (6)	
H20D	0.457484	0.576027	0.491127	0.070*	
H20E	0.380958	0.627181	0.512244	0.070*	
H20F	0.474669	0.622809	0.587577	0.070*	

### Atomic displacement parameters (Å^2^)

**Table:**

	*U*^11^	*U*^22^	*U*^33^	*U*^12^	*U*^13^	*U*^23^
O1	0.0145 (5)	0.0188 (6)	0.0201 (6)	−0.0002 (4)	0.0048 (5)	−0.0043 (5)
O2A	0.0220 (6)	0.0240 (6)	0.0192 (6)	0.0064 (5)	0.0082 (5)	0.0051 (5)
O2B	0.0190 (6)	0.0296 (7)	0.0381 (8)	0.0035 (5)	0.0169 (5)	0.0044 (6)
O3B	0.0151 (6)	0.0332 (7)	0.0390 (8)	−0.0048 (5)	0.0092 (5)	−0.0001 (6)
N2A	0.0159 (6)	0.0148 (7)	0.0180 (7)	−0.0012 (5)	0.0056 (5)	−0.0014 (6)
N1B	0.0144 (6)	0.0164 (7)	0.0169 (7)	−0.0012 (5)	0.0078 (5)	−0.0029 (6)
N2B	0.0136 (6)	0.0184 (7)	0.0174 (7)	−0.0029 (5)	0.0054 (5)	−0.0025 (6)
N1A	0.0144 (6)	0.0170 (7)	0.0166 (7)	0.0009 (5)	0.0037 (5)	−0.0004 (6)
O3A	0.0296 (7)	0.0184 (6)	0.0426 (8)	0.0034 (5)	0.0115 (6)	0.0083 (6)
C8A	0.0146 (8)	0.0184 (9)	0.0215 (9)	0.0006 (6)	0.0074 (7)	−0.0020 (7)
C1B	0.0175 (8)	0.0163 (8)	0.0143 (8)	0.0010 (6)	0.0075 (6)	0.0024 (6)
C4A	0.0181 (8)	0.0260 (9)	0.0185 (9)	−0.0018 (7)	0.0026 (7)	−0.0026 (7)
C14B	0.0143 (7)	0.0201 (9)	0.0129 (8)	−0.0014 (6)	0.0056 (6)	−0.0031 (6)
C19B	0.0219 (8)	0.0185 (9)	0.0194 (9)	−0.0011 (7)	0.0085 (7)	0.0003 (7)
C11A	0.0158 (8)	0.0206 (9)	0.0184 (9)	0.0014 (6)	0.0067 (7)	−0.0016 (7)
C7B	0.0140 (7)	0.0143 (8)	0.0173 (8)	−0.0023 (6)	0.0055 (6)	−0.0055 (6)
C12B	0.0179 (8)	0.0156 (8)	0.0176 (9)	0.0014 (6)	0.0054 (7)	0.0004 (7)
C9B	0.0169 (8)	0.0198 (9)	0.0196 (9)	0.0018 (6)	0.0035 (7)	−0.0004 (7)
C2A	0.0159 (8)	0.0167 (8)	0.0190 (9)	−0.0021 (6)	0.0095 (7)	−0.0026 (7)
C8B	0.0194 (8)	0.0187 (8)	0.0167 (9)	−0.0031 (7)	0.0072 (7)	−0.0009 (7)
C7A	0.0158 (8)	0.0135 (8)	0.0163 (8)	−0.0023 (6)	0.0027 (6)	−0.0024 (7)
C12A	0.0186 (8)	0.0204 (9)	0.0161 (8)	−0.0013 (7)	0.0082 (7)	−0.0009 (7)
C11B	0.0233 (8)	0.0182 (9)	0.0207 (9)	−0.0022 (7)	0.0121 (7)	−0.0006 (7)
C10A	0.0186 (8)	0.0154 (8)	0.0150 (8)	−0.0001 (6)	0.0041 (7)	−0.0013 (7)
C9A	0.0206 (8)	0.0210 (9)	0.0184 (9)	−0.0018 (7)	0.0101 (7)	−0.0004 (7)
C10B	0.0157 (8)	0.0171 (8)	0.0257 (9)	−0.0008 (6)	0.0092 (7)	−0.0032 (7)
C2B	0.0169 (8)	0.0141 (8)	0.0143 (8)	−0.0015 (6)	0.0043 (6)	0.0018 (6)
C5A	0.0189 (8)	0.0205 (9)	0.0246 (9)	−0.0062 (7)	0.0063 (7)	−0.0063 (7)
C15B	0.0162 (8)	0.0204 (9)	0.0207 (9)	0.0034 (6)	0.0067 (7)	0.0020 (7)
C4B	0.0257 (9)	0.0208 (9)	0.0211 (9)	0.0021 (7)	0.0116 (7)	−0.0016 (7)
C6A	0.0172 (8)	0.0171 (8)	0.0219 (9)	−0.0009 (6)	0.0083 (7)	0.0004 (7)
C18B	0.0163 (8)	0.0264 (9)	0.0161 (9)	0.0054 (7)	0.0036 (7)	0.0005 (7)
C15A	0.0164 (8)	0.0207 (9)	0.0220 (9)	−0.0020 (6)	0.0077 (7)	−0.0003 (7)
C13A	0.0222 (9)	0.0271 (10)	0.0202 (9)	0.0070 (7)	0.0064 (7)	0.0008 (7)
C16B	0.0216 (8)	0.0177 (9)	0.0275 (10)	−0.0019 (7)	0.0108 (7)	−0.0002 (7)
C17B	0.0148 (8)	0.0268 (9)	0.0181 (9)	−0.0042 (7)	0.0068 (7)	−0.0051 (7)
C17A	0.0247 (9)	0.0160 (8)	0.0236 (9)	0.0009 (7)	0.0075 (7)	0.0005 (7)
C5B	0.0225 (8)	0.0198 (9)	0.0184 (9)	−0.0041 (7)	0.0063 (7)	−0.0036 (7)
C19A	0.0162 (8)	0.0212 (9)	0.0305 (10)	0.0014 (7)	0.0097 (7)	0.0009 (8)
C3A	0.0173 (8)	0.0217 (9)	0.0190 (9)	0.0010 (7)	0.0060 (7)	0.0014 (7)
C14A	0.0191 (8)	0.0159 (8)	0.0153 (8)	0.0000 (6)	0.0060 (7)	−0.0025 (7)
C3B	0.0169 (8)	0.0223 (9)	0.0194 (9)	−0.0008 (7)	0.0088 (7)	0.0006 (7)
C6B	0.0153 (8)	0.0178 (9)	0.0188 (9)	−0.0025 (6)	0.0065 (7)	0.0004 (7)
C1A	0.0136 (7)	0.0199 (9)	0.0172 (9)	−0.0006 (6)	0.0067 (7)	−0.0018 (7)
B1B	0.0160 (9)	0.0152 (9)	0.0146 (9)	0.0000 (7)	0.0045 (7)	0.0011 (7)
C16A	0.0175 (8)	0.0217 (9)	0.0239 (9)	0.0034 (7)	0.0066 (7)	−0.0018 (7)
C18A	0.0214 (8)	0.0222 (9)	0.0372 (11)	−0.0038 (7)	0.0130 (8)	0.0027 (8)
B1A	0.0137 (8)	0.0197 (10)	0.0180 (10)	−0.0005 (7)	0.0081 (7)	−0.0041 (8)
C13B	0.0304 (10)	0.0370 (12)	0.0588 (14)	0.0033 (8)	0.0320 (10)	0.0105 (10)
C20A	0.0372 (11)	0.0230 (10)	0.0457 (13)	0.0108 (8)	0.0112 (9)	0.0073 (9)
C20B	0.0231 (10)	0.0426 (13)	0.0723 (17)	−0.0100 (9)	0.0155 (10)	0.0125 (12)

### Geometric parameters (Å, º)

**Table:**

O1—B1B	1.368 (2)	C7A—C12A	1.384 (2)
O1—B1A	1.372 (2)	C12A—H12A	0.9500
O2A—C10A	1.3673 (18)	C11B—H11B	0.9500
O2A—C13A	1.4272 (18)	C11B—C10B	1.384 (2)
O2B—C10B	1.3730 (18)	C10A—C9A	1.394 (2)
O2B—C13B	1.430 (2)	C9A—H9A	0.9500
O3B—C17B	1.3757 (18)	C2B—C3B	1.384 (2)
O3B—C20B	1.422 (2)	C5A—H5A	0.9500
N2A—C2A	1.4124 (19)	C5A—C6A	1.392 (2)
N2A—C14A	1.427 (2)	C15B—H15B	0.9500
N2A—B1A	1.437 (2)	C15B—C16B	1.392 (2)
N1B—C1B	1.4040 (19)	C4B—H4B	0.9500
N1B—C7B	1.4407 (19)	C4B—C5B	1.391 (2)
N1B—B1B	1.432 (2)	C4B—C3B	1.393 (2)
N2B—C14B	1.4322 (19)	C6A—H6A	0.9500
N2B—C2B	1.4079 (19)	C6A—C1A	1.383 (2)
N2B—B1B	1.433 (2)	C18B—H18B	0.9500
N1A—C7A	1.431 (2)	C18B—C17B	1.388 (2)
N1A—C1A	1.4034 (19)	C15A—H15A	0.9500
N1A—B1A	1.428 (2)	C15A—C14A	1.386 (2)
O3A—C17A	1.3720 (19)	C15A—C16A	1.389 (2)
O3A—C20A	1.425 (2)	C13A—H13A	0.9800
C8A—H8A	0.9500	C13A—H13B	0.9800
C8A—C7A	1.393 (2)	C13A—H13C	0.9800
C8A—C9A	1.378 (2)	C16B—H16B	0.9500
C1B—C2B	1.410 (2)	C16B—C17B	1.386 (2)
C1B—C6B	1.379 (2)	C17A—C16A	1.388 (2)
C4A—H4A	0.9500	C17A—C18A	1.390 (2)
C4A—C5A	1.386 (2)	C5B—H5B	0.9500
C4A—C3A	1.395 (2)	C5B—C6B	1.395 (2)
C14B—C19B	1.390 (2)	C19A—H19A	0.9500
C14B—C15B	1.383 (2)	C19A—C14A	1.389 (2)
C19B—H19B	0.9500	C19A—C18A	1.383 (2)
C19B—C18B	1.380 (2)	C3A—H3A	0.9500
C11A—H11A	0.9500	C3B—H3B	0.9500
C11A—C12A	1.388 (2)	C6B—H6B	0.9500
C11A—C10A	1.385 (2)	C16A—H16A	0.9500
C7B—C12B	1.381 (2)	C18A—H18A	0.9500
C7B—C8B	1.391 (2)	C13B—H13D	0.9800
C12B—H12B	0.9500	C13B—H13E	0.9800
C12B—C11B	1.392 (2)	C13B—H13F	0.9800
C9B—H9B	0.9500	C20A—H20A	0.9800
C9B—C8B	1.380 (2)	C20A—H20B	0.9800
C9B—C10B	1.391 (2)	C20A—H20C	0.9800
C2A—C3A	1.379 (2)	C20B—H20D	0.9800
C2A—C1A	1.407 (2)	C20B—H20E	0.9800
C8B—H8B	0.9500	C20B—H20F	0.9800
			
B1B—O1—B1A	132.75 (13)	C5B—C4B—C3B	121.34 (15)
C10A—O2A—C13A	116.62 (12)	C3B—C4B—H4B	119.3
C10B—O2B—C13B	116.33 (13)	C5A—C6A—H6A	121.2
C17B—O3B—C20B	118.15 (13)	C1A—C6A—C5A	117.54 (15)
C2A—N2A—C14A	123.34 (13)	C1A—C6A—H6A	121.2
C2A—N2A—B1A	107.38 (13)	C19B—C18B—H18B	119.9
C14A—N2A—B1A	129.26 (13)	C19B—C18B—C17B	120.13 (15)
C1B—N1B—C7B	122.71 (12)	C17B—C18B—H18B	119.9
C1B—N1B—B1B	107.83 (12)	C14A—C15A—H15A	119.4
B1B—N1B—C7B	129.45 (13)	C14A—C15A—C16A	121.25 (15)
C14B—N2B—B1B	129.55 (13)	C16A—C15A—H15A	119.4
C2B—N2B—C14B	122.27 (12)	O2A—C13A—H13A	109.5
C2B—N2B—B1B	107.94 (12)	O2A—C13A—H13B	109.5
C1A—N1A—C7A	121.93 (13)	O2A—C13A—H13C	109.5
C1A—N1A—B1A	108.04 (13)	H13A—C13A—H13B	109.5
B1A—N1A—C7A	130.03 (13)	H13A—C13A—H13C	109.5
C17A—O3A—C20A	117.01 (13)	H13B—C13A—H13C	109.5
C7A—C8A—H8A	119.8	C15B—C16B—H16B	120.6
C9A—C8A—H8A	119.8	C17B—C16B—C15B	118.85 (15)
C9A—C8A—C7A	120.39 (14)	C17B—C16B—H16B	120.6
N1B—C1B—C2B	108.82 (13)	O3B—C17B—C18B	115.02 (14)
C6B—C1B—N1B	130.41 (14)	O3B—C17B—C16B	124.66 (15)
C6B—C1B—C2B	120.74 (14)	C16B—C17B—C18B	120.30 (14)
C5A—C4A—H4A	119.4	O3A—C17A—C16A	124.35 (14)
C5A—C4A—C3A	121.27 (15)	O3A—C17A—C18A	116.17 (14)
C3A—C4A—H4A	119.4	C16A—C17A—C18A	119.47 (15)
C19B—C14B—N2B	120.81 (14)	C4B—C5B—H5B	119.6
C15B—C14B—N2B	120.11 (13)	C4B—C5B—C6B	120.83 (15)
C15B—C14B—C19B	119.08 (14)	C6B—C5B—H5B	119.6
C14B—C19B—H19B	119.8	C14A—C19A—H19A	119.5
C18B—C19B—C14B	120.34 (15)	C18A—C19A—H19A	119.5
C18B—C19B—H19B	119.8	C18A—C19A—C14A	120.92 (15)
C12A—C11A—H11A	120.2	C4A—C3A—H3A	120.9
C10A—C11A—H11A	120.2	C2A—C3A—C4A	118.20 (15)
C10A—C11A—C12A	119.59 (14)	C2A—C3A—H3A	120.9
C12B—C7B—N1B	120.36 (14)	C15A—C14A—N2A	121.32 (14)
C12B—C7B—C8B	119.32 (14)	C15A—C14A—C19A	118.41 (15)
C8B—C7B—N1B	120.32 (14)	C19A—C14A—N2A	120.25 (13)
C7B—C12B—H12B	119.5	C2B—C3B—C4B	117.73 (14)
C7B—C12B—C11B	120.99 (15)	C2B—C3B—H3B	121.1
C11B—C12B—H12B	119.5	C4B—C3B—H3B	121.1
C8B—C9B—H9B	119.7	C1B—C6B—C5B	118.25 (14)
C8B—C9B—C10B	120.55 (15)	C1B—C6B—H6B	120.9
C10B—C9B—H9B	119.7	C5B—C6B—H6B	120.9
C3A—C2A—N2A	130.98 (15)	N1A—C1A—C2A	108.66 (13)
C3A—C2A—C1A	120.25 (14)	C6A—C1A—N1A	129.58 (14)
C1A—C2A—N2A	108.63 (13)	C6A—C1A—C2A	121.68 (14)
C7B—C8B—H8B	120.0	O1—B1B—N1B	124.79 (14)
C9B—C8B—C7B	119.98 (15)	O1—B1B—N2B	128.08 (14)
C9B—C8B—H8B	120.0	N1B—B1B—N2B	107.10 (14)
C8A—C7A—N1A	120.37 (13)	C15A—C16A—H16A	120.1
C12A—C7A—N1A	120.31 (14)	C17A—C16A—C15A	119.70 (14)
C12A—C7A—C8A	119.31 (14)	C17A—C16A—H16A	120.1
C11A—C12A—H12A	119.6	C17A—C18A—H18A	119.9
C7A—C12A—C11A	120.70 (15)	C19A—C18A—C17A	120.19 (15)
C7A—C12A—H12A	119.6	C19A—C18A—H18A	119.9
C12B—C11B—H11B	120.3	O1—B1A—N2A	127.87 (15)
C10B—C11B—C12B	119.39 (15)	O1—B1A—N1A	124.76 (15)
C10B—C11B—H11B	120.3	N1A—B1A—N2A	107.27 (13)
O2A—C10A—C11A	124.14 (14)	O2B—C13B—H13D	109.5
O2A—C10A—C9A	115.80 (14)	O2B—C13B—H13E	109.5
C11A—C10A—C9A	120.06 (15)	O2B—C13B—H13F	109.5
C8A—C9A—C10A	119.91 (15)	H13D—C13B—H13E	109.5
C8A—C9A—H9A	120.0	H13D—C13B—H13F	109.5
C10A—C9A—H9A	120.0	H13E—C13B—H13F	109.5
O2B—C10B—C9B	115.73 (14)	O3A—C20A—H20A	109.5
O2B—C10B—C11B	124.52 (15)	O3A—C20A—H20B	109.5
C11B—C10B—C9B	119.75 (14)	O3A—C20A—H20C	109.5
N2B—C2B—C1B	108.31 (13)	H20A—C20A—H20B	109.5
C3B—C2B—N2B	130.53 (14)	H20A—C20A—H20C	109.5
C3B—C2B—C1B	121.12 (14)	H20B—C20A—H20C	109.5
C4A—C5A—H5A	119.5	O3B—C20B—H20D	109.5
C4A—C5A—C6A	121.04 (15)	O3B—C20B—H20E	109.5
C6A—C5A—H5A	119.5	O3B—C20B—H20F	109.5
C14B—C15B—H15B	119.4	H20D—C20B—H20E	109.5
C14B—C15B—C16B	121.26 (14)	H20D—C20B—H20F	109.5
C16B—C15B—H15B	119.4	H20E—C20B—H20F	109.5
C5B—C4B—H4B	119.3		
			
O2A—C10A—C9A—C8A	178.55 (14)	C2B—N2B—B1B—N1B	−0.61 (17)
N2A—C2A—C3A—C4A	175.54 (15)	C2B—C1B—C6B—C5B	−0.2 (2)
N2A—C2A—C1A—N1A	0.14 (17)	C5A—C4A—C3A—C2A	0.3 (2)
N2A—C2A—C1A—C6A	−177.10 (14)	C5A—C6A—C1A—N1A	−175.77 (15)
N1B—C1B—C2B—N2B	0.47 (17)	C5A—C6A—C1A—C2A	0.8 (2)
N1B—C1B—C2B—C3B	178.43 (14)	C15B—C14B—C19B—C18B	1.4 (2)
N1B—C1B—C6B—C5B	−177.82 (15)	C15B—C16B—C17B—O3B	−176.14 (15)
N1B—C7B—C12B—C11B	−178.68 (14)	C15B—C16B—C17B—C18B	2.2 (2)
N1B—C7B—C8B—C9B	179.28 (14)	C4B—C5B—C6B—C1B	−0.3 (2)
N2B—C14B—C19B—C18B	−178.20 (14)	C13A—O2A—C10A—C11A	13.0 (2)
N2B—C14B—C15B—C16B	179.40 (14)	C13A—O2A—C10A—C9A	−167.12 (14)
N2B—C2B—C3B—C4B	177.41 (15)	C5B—C4B—C3B—C2B	−0.4 (2)
N1A—C7A—C12A—C11A	179.14 (14)	C3A—C4A—C5A—C6A	−0.3 (2)
O3A—C17A—C16A—C15A	177.38 (16)	C3A—C2A—C1A—N1A	176.32 (14)
O3A—C17A—C18A—C19A	−176.64 (16)	C3A—C2A—C1A—C6A	−0.9 (2)
C8A—C7A—C12A—C11A	−1.7 (2)	C14A—N2A—C2A—C3A	6.7 (3)
C1B—N1B—C7B—C12B	−100.79 (18)	C14A—N2A—C2A—C1A	−177.71 (13)
C1B—N1B—C7B—C8B	78.92 (19)	C14A—N2A—B1A—O1	−6.6 (3)
C1B—N1B—B1B—O1	−177.24 (15)	C14A—N2A—B1A—N1A	177.07 (14)
C1B—N1B—B1B—N2B	0.90 (17)	C14A—C15A—C16A—C17A	−0.5 (3)
C1B—C2B—C3B—C4B	0.0 (2)	C14A—C19A—C18A—C17A	−1.1 (3)
C4A—C5A—C6A—C1A	−0.2 (2)	C3B—C4B—C5B—C6B	0.6 (3)
C14B—N2B—C2B—C1B	174.97 (13)	C6B—C1B—C2B—N2B	−177.64 (14)
C14B—N2B—C2B—C3B	−2.7 (3)	C6B—C1B—C2B—C3B	0.3 (2)
C14B—N2B—B1B—O1	3.1 (3)	C1A—N1A—C7A—C8A	−78.00 (19)
C14B—N2B—B1B—N1B	−174.99 (14)	C1A—N1A—C7A—C12A	101.15 (17)
C14B—C19B—C18B—C17B	−0.8 (2)	C1A—N1A—B1A—O1	−175.29 (15)
C14B—C15B—C16B—C17B	−1.6 (2)	C1A—N1A—B1A—N2A	1.21 (17)
C19B—C14B—C15B—C16B	−0.2 (2)	C1A—C2A—C3A—C4A	0.3 (2)
C19B—C18B—C17B—O3B	177.45 (14)	B1B—O1—B1A—N2A	62.9 (3)
C19B—C18B—C17B—C16B	−1.0 (2)	B1B—O1—B1A—N1A	−121.33 (19)
C11A—C10A—C9A—C8A	−1.5 (2)	B1B—N1B—C1B—C2B	−0.85 (17)
C7B—N1B—C1B—C2B	−179.69 (13)	B1B—N1B—C1B—C6B	177.02 (16)
C7B—N1B—C1B—C6B	−1.8 (2)	B1B—N1B—C7B—C12B	80.6 (2)
C7B—N1B—B1B—O1	1.5 (3)	B1B—N1B—C7B—C8B	−99.6 (2)
C7B—N1B—B1B—N2B	179.63 (14)	B1B—N2B—C14B—C19B	−119.01 (18)
C7B—C12B—C11B—C10B	−0.9 (2)	B1B—N2B—C14B—C15B	61.4 (2)
C12B—C7B—C8B—C9B	−1.0 (2)	B1B—N2B—C2B—C1B	0.10 (17)
C12B—C11B—C10B—O2B	179.54 (14)	B1B—N2B—C2B—C3B	−177.61 (17)
C12B—C11B—C10B—C9B	−0.4 (2)	C16A—C15A—C14A—N2A	−179.44 (15)
C2A—N2A—C14A—C15A	47.6 (2)	C16A—C15A—C14A—C19A	1.9 (2)
C2A—N2A—C14A—C19A	−133.79 (16)	C16A—C17A—C18A—C19A	2.5 (3)
C2A—N2A—B1A—O1	175.24 (15)	C18A—C17A—C16A—C15A	−1.7 (3)
C2A—N2A—B1A—N1A	−1.12 (17)	C18A—C19A—C14A—N2A	−179.75 (16)
C8B—C7B—C12B—C11B	1.6 (2)	C18A—C19A—C14A—C15A	−1.1 (3)
C8B—C9B—C10B—O2B	−178.96 (14)	B1A—O1—B1B—N1B	−167.79 (16)
C8B—C9B—C10B—C11B	1.0 (2)	B1A—O1—B1B—N2B	14.5 (3)
C7A—N1A—C1A—C2A	178.84 (13)	B1A—N2A—C2A—C3A	−175.02 (16)
C7A—N1A—C1A—C6A	−4.2 (2)	B1A—N2A—C2A—C1A	0.61 (17)
C7A—N1A—B1A—O1	5.1 (3)	B1A—N2A—C14A—C15A	−130.34 (17)
C7A—N1A—B1A—N2A	−178.45 (14)	B1A—N2A—C14A—C19A	48.3 (2)
C7A—C8A—C9A—C10A	0.6 (2)	B1A—N1A—C7A—C8A	101.61 (19)
C12A—C11A—C10A—O2A	−179.27 (14)	B1A—N1A—C7A—C12A	−79.2 (2)
C12A—C11A—C10A—C9A	0.8 (2)	B1A—N1A—C1A—C2A	−0.85 (17)
C10A—C11A—C12A—C7A	0.8 (2)	B1A—N1A—C1A—C6A	176.11 (16)
C9A—C8A—C7A—N1A	−179.86 (14)	C13B—O2B—C10B—C9B	179.79 (15)
C9A—C8A—C7A—C12A	1.0 (2)	C13B—O2B—C10B—C11B	−0.2 (2)
C10B—C9B—C8B—C7B	−0.3 (2)	C20A—O3A—C17A—C16A	17.1 (2)
C2B—N2B—C14B—C19B	67.3 (2)	C20A—O3A—C17A—C18A	−163.81 (16)
C2B—N2B—C14B—C15B	−112.25 (17)	C20B—O3B—C17B—C18B	−175.17 (17)
C2B—N2B—B1B—O1	177.44 (16)	C20B—O3B—C17B—C16B	3.2 (3)

### Hydrogen-bond geometry (Å, º)

**Table:**

*D*—H···*A*	*D*—H	H···*A*	*D*···*A*	*D*—H···*A*
C8*B*—H8*B*···O2*A*^i^	0.95	2.40	3.233 (2)	147
C13*B*—H13*E*···O3*B*^ii^	0.98	2.46	3.374 (3)	155

Symmetry codes: (i) −*x*+2, *y*+1/2, −*z*+3/2; (ii) *x*+1, *y*, *z*.
